# Sex‐specific alterations in NOS regulation of vascular function in aorta and mesenteric arteries from spontaneously hypertensive rats compared to Wistar Kyoto rats

**DOI:** 10.14814/phy2.12125

**Published:** 2014-08-28

**Authors:** Analia S. Loria, Krystal N. Brinson, Brandon M. Fox, Jennifer C. Sullivan

**Affiliations:** 1 Department of Pharmacology and Nutritional Sciences, University of Kentucky, St. Lexington, Kentucky; 2 Department of Physiology, Georgia Regents University, Augusta, Georgia

**Keywords:** NOS activity, NOS expression, phenylephrine, SHR

## Abstract

The present study tested the hypothesis that spontaneously hypertensive rats (SHR) have impaired nitric oxide synthase (NOS)‐mediated regulation of vascular function versus Wistar‐Kyoto rats (WKY). Aorta and small mesenteric arteries were studied from male and female SHR (M SHR and F SHR) and WKY (M WKY and F WKY). Phenylephrine (PE)‐induced vasoconstriction was greater in aorta of M SHR versus all others (*P* < 0.05); there were neither sex nor strain differences in PE contraction in mesenteric arteries. The NOS inhibitor l‐Nitro‐Arginine Methyl Ester (l‐NAME) increased PE‐induced vasoconstriction in all rats, although the increase was the least in male SHR (*P* < 0.05), revealing a blunted vasoconstrictor buffering capacity of NOS. l‐NAME increased sensitivity to PE‐induced constriction only in mesenteric arteries of SHR, although, the maximal percent increase in contraction was comparable among groups. ACh‐induced relaxation was also less in aorta from M SHR versus all others (*P* < 0.05). ACh relaxation was comparable among groups in mesenteric arteries, although SHR exhibited a greater NOS component to ACh‐induced relaxation than WKY. To gain mechanistic insight into sex and strain differences in vascular function, NOS activity and NOS3 protein expression were measured. Aortic NOS activity was comparable between groups and M SHR had greater NOS3 expression than M WKY. In contrast, although vascular function was largely maintained in mesenteric arteries of SHR, NOS activity was less in SHR versus WKY. In conclusion, M SHR exhibit a decrease in NOS regulation of vascular function compared to F SHR and WKY, although this is not mediated by decreases in NOS activity and/or expression.

## Abstract (precis)

The present study tested the hypothesis that spontaneously hypertensive rats (SHR) have impaired nitric oxide synthase (NOS)‐mediated regulation of vascular function versus Wistar‐Kyoto rats (WKY). Aorta and small mesenteric arteries were studied from male and female SHR and WKY. Male SHR showed a decreased NOS regulation of vascular function compared to F SHR and WKY, although this was not mediated by decreases in NOS activity and/or expression.

## Introduction

Men have a greater incidence and severity of cardiovascular disease compared to women until menopause (Gerhard and Ganz 1995; Wiinberg et al. 1995). A similar sex disparity in cardiovascular disease is seen in a number of experimental animal models of hypertension, including spontaneously hypertensive rats (SHR) (Kauser and Rubanyi 1995; Iliescu et al. 2006). SHR are a genetic model of hypertension and, similar to the human population, male SHR have a more rapid increase in blood pressure over time compared to female SHR. Although the molecular mechanisms responsible for sex differences in cardiovascular health and function are still unclear, the nitric oxide (NO)/NO synthase (NOS) pathway has been implicated in humans (Forte et al. 1998; Sader and Celermajer 2002) and rats (Baylis et al. 1992; Ribeiro et al. 1992; Kauser and Rubanyi 1994, Gamboa et al. 2007) and we recently published that female SHR are more dependent on NOS to maintain their blood pressure than males (Brinson et al. 2013).

NO is a key molecule regulating endothelial and vascular function (Forte et al. 1998; Vaziri et al. 1998). In particular, loss of NOS‐mediated control of vascular function contributes to both the development and progression of cardiovascular disease (Anishchenko and Igosheva 1992; McIntyre et al. 1997; Sader and Celermajer 2002; Orshal and Khalil 2004). It is also well established that there are sex differences in NO bioavailability, with females having greater levels of NO than males (Forte et al. 1998; Glushkovskaya‐Semyachkina et al. 2006; Sullivan et al. 2010) and numerous studies have demonstrated that greater NO in females contributes to maintaining cardiovascular function with age and disease relative to males (McIntyre et al. 1997; Miller 2010; Baylis 2012). However, whether there is differential NOS regulation of vasoconstriction and endothelial function in small and large arteries between males and females with normal blood pressure or hypertension has not been fully determined.

The impact of NOS on vascular function may be dependent, in part, on the size and physiological role of the artery. Decreases in NO bioavailability and NOS‐mediated vasorelaxation are commonly noted in conduit arteries from male animal models of hypertension, although we previously reported that NOS‐mediated vasorelaxation was maintained in small arteries from male hypertensive rats (Sasser et al. 2004; Kang et al. 2007). This finding is despite the fact that we and others have previously shown that endothelial NOS (NOS3; eNOS) protein expression is unchanged or even increased in the vasculature in hypertensive male animal models including SHR (Vaziri et al. 1998; Piech et al. 2003; Sun et al. 2013).

Therefore, the present study was designed to test the hypothesis that SHR have impaired NOS‐mediated regulation of vascular function (both constrictive and vasorelaxant) and reduced NOS activity and expression versus Wistar‐Kyoto rats (WKY). However, we further hypothesize that female SHR will maintain greater NOS‐mediated control of vascular function relative to males due to greater levels of NO in female SHR. Studies employed aorta, a conduit artery, and small mesenteric arteries and the relative impact of NOS on both vasoconstriction and vasodilation were measured in male and female SHR and WKY.

## Methods

### Animals

Male and female SHR and WKY (Harlan Laboratories, Indianapolis, IN) were studied. All experiments were conducted in accordance with the National Institutes of Health Guide for the Care and Use of Laboratory Animals, European Union Directive 2010/63/EU for animal experiments and approved and monitored by the Georgia Regents University IACUC. Rats were housed in temperature and humidity‐controlled, light‐cycled quarters and maintained on a standard rodent diet (Teklad 8604, Madison, WI). At 12–13 weeks of age, rats were anesthetized with ketamine/xylazine (50 mg/kg and 6 mg/kg ip, respectively; Phoenix Pharmaceuticals, St. Joseph, MO) and thoracic aortas and third‐order mesenteric arteries were isolated.

### Vascular reactivity

Thoracic aortas were cleaned of adherent adipose tissue, cut into concentric rings, and mounted on pins for wire myography (Danish Myo Technology A/S, Aarhus, Denmark) as previously described (Sullivan et al. 2004; Loria et al. 2011). Similarly, third‐order mesenteric arteries were isolated, cleaned of adherent adipose tissue, cut into segments, and mounted on wires for myography as previously described (Sullivan et al. 2004). Aortas preload tension was fixed to 28 mN, while mesenteric arteries preload tension was 4 mN. Vessel segments were equilibrated for 30 min before the viability of the vessel was determined by a robust vasoconstrictor response to 1 *μ*mol/L phenylephrine (PE) followed by vasorelaxation to 10 *μ*mol/L acetylcholine (ACh); only arteries that relaxed at least 80% of the maximal PE‐induced constriction were included in the study. Drugs were rinsed out and the vessels were allowed to reequilibrate for 30 min. Vasoconstriction was assessed by performing cumulative concentration–response (CCR) curves to the *α*‐adrenergic agonist PE (1 × 10^−9^–3 × 10^−5^ mol/L) in the absence or presence of the nonselective NOS inhibitor l‐Nitro‐Arginine Methyl Ester (l‐NAME, 15 min preincubation at 100 *μ*mol/L, Sigma). The ability of NO to attenuate PE‐induced constriction, or the vasoconstrictor buffering capacity of NOS (Loria et al. 2011), was calculated as the delta of area under the curve (AUC, % increase in force × mol/L), determined by the difference in PE‐induced AUC ± l‐NAME. Vessel segments were then rinsed, allowed to reequilibrate and CCR curves were performed to KCl (8 × 10^−3^ to 100 × 10^−3^ mol/L) in the same artery segments. Vasoconstriction data are presented as percent increase in force from baseline tension. Endothelial‐dependent relaxation was also assessed in aortic and small mesenteric artery segments following preconstriction with 1 *μ*mol/L or 2 *μ*mol/L PE, respectively, followed by CCR curves to acetylcholine (ACh; 1 × 10^−9^–1 × 10^−5^ mol/L) in the absence or presence of the l‐NAME (15 min preincubation at 100 *μ*mol/L). Accordingly, delta AUC (% relaxation × mol/L) was determined by the difference in ACh‐induced AUC ± l‐NAME. There were no differences in the level of precontraction between the different rat groups. CCR curves were then performed to the endothelium‐independent vasodilator sodium nitroprusside (SNP; 100 pmol/L to 31.6 *μ*mol/L) in the same artery segments. Vasorelaxation data are presented as percent relaxation from PE‐induced constriction. For all CCR, maximum response and sensitivity to the vasoactive agonists, pD_2_ (‐logEC50), were calculated.

### NOS activity assay

Vascular tissue (whole aorta and mesenteric arterial beds) were isolated from additional rats and homogenized as previously described (Sullivan et al. 2010). Briefly, tissue was snap‐frozen and homogenized in cold homogenization buffer (20.0 mmol/L Tris·HCl pH 7.4, 137.0 mmol/L NaCl, 10% glycerol, and 1% NP40) containing protease inhibitors (1 mmol/L PMSF, 1 *μ*mol/L pepstatin A, 2 *μ*mol/L leupeptin, and 0.1% aprotinin). To determine total NOS enzymatic activity, tissue homogenates were incubated with [^3^H]arginine (10 *μ*mol/L final arginine, 71 Ci/mmol) in the presence of excess cofactors, as previously described, in a final volume of 50 *μ*L for 30 min at room temperature (Sullivan et al. 2010; Loria et al. 2011). NOS activity was normalized to total protein concentration and expressed as pmoles citrulline/mg protein/30 min. Protein concentrations were determined by standard Bradford assay (Bio‐Rad, Hercules, CA) with the use of BSA as the standard.

### Western blot analysis

Western blotting was performed as previously described using the same vessel homogenates that were used in the NOS activity measurement (Sullivan et al. 2002, 2004). Two‐color immunoblots were analyzed using polyclonal primary antibodies to NOS3 (1:500; BD Biosciences, San Jose, CA) and *β*‐actin (A1978, 1:10,000; Sigma, St Louis, MO). After incubation with secondary antibody (IRDye 800‐conjugated affinity‐purified anti‐mouse IgG, 1:2,000 or AlexaFluor 680‐conjugated affinity‐purified anti‐rabbit IgG, 1:2,000), the blot was scanned and the intensity of specific bands were quantified using the Odyssey Infrared Imaging System (LI‐COR Biosciences, Lincoln, NE). Densitometric results were reported normalized to *β*‐actin.

### Data analysis

All values are expressed as mean ± SEM. Percent increase in force or relaxation in arteries, pD_2_ values, vasoconstrictor buffering capacity of NOS, and NOS activity in male and female SHR and WKY were compared using a Two‐way ANOVA with Bonferroni's post hoc performed using GraphPad Prism version 5.01 for Windows (GraphPad Software, San Diego, CA). NOS3 protein expression maximal response and pD_2_ values in control and l‐NAME‐treated vessels within each group were compared using a Student's *t*‐test. For all comparisons, a value of *P* < 0.05 was considered statistically significant.

## Results

### Sex and strain differences in PE‐induced constriction and NOS capacity

**Figure 1.**
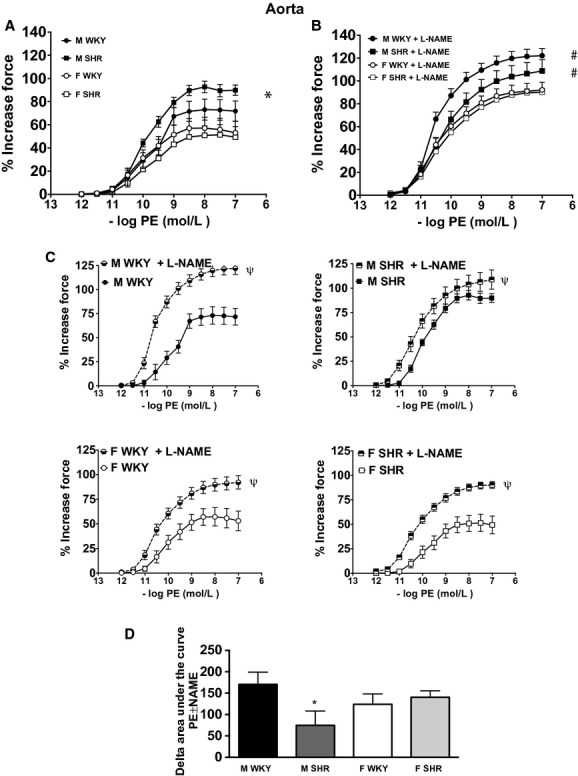
Vasoconstriction induced by PE in aorta from male and female Wistar‐Kyoto rats (WKY) and spontaneously hypertensive rats (SHR) in the absence (A) or presence (B) of l‐Nitro‐Arginine Methyl Ester (l‐NAME). Panel C illustrates the impact of l‐NAME on PE‐induced constriction within each group. Panel D illustrates the ability of NO to attenuate PE‐induced constriction, or the vasoconstrictor buffering capacity of nitric oxide synthase (NOS), which was calculated as the change in the area under the curve in the presence or absence of l‐NAME (% increase in force × mol/L). *indicates significant difference in maximal % increase in force versus all other groups, *P* < 0.05. ^#^indicates significant differences in maximal % increase in force versus female rats, *P* < 0.05. ^*ψ*^indicates significant differences in maximal increase in force versus nontreated control rings, *P* < 0.05; *N* = 5–7.

Alpha‐adrenergic‐induced vasoconstriction was assessed in isolated aorta and mesenteric arteries from male and female SHR and WKY. PE‐induced vasoconstriction was not significantly different in aortic rings from male and female WKY (Table 1 and Fig. 1A). However, PE‐induced vasoconstriction was greater in aortic rings from male SHR compared with both female SHR and male and female WKY. PE‐induced constriction was comparable in female SHR and WKY (effect of sex = *P* < 0.05; effect of strain = NS; interaction = *P* < 0.05). Additional studies assessed the ability of basal NO levels to attenuate PE‐induced constriction in the aorta. Preincubation with l‐NAME increased maximal constriction to PE in all groups (Fig. 1B). The individual comparisons are depicted in panel 1C. Vasoconstrictor buffering capacity of NOS was significantly less in male SHR than in all other groups (effect of sex = NS; effect of strain = NS; interaction *P* < 0.05, Fig. 1D).

**Table 1.** Sensitivity and maximal responses to PE, ACh, SNP and KCl in absence or presence of a total nitric oxide synthase (NOS) inhibitor (l‐NAME).

	Male WKY		Male SHR		Female WKY		Females SHR	
	Aorta	Mesenteric	Aorta	Mesenteric	Aorta	Mesenteric	Aorta	Mesenteric
(A)								
Treatment	% Increase Force	% Increase Force	% Increase Force	% Increase Force	% Increase Force	% Increase Force	% Increase Force	% Increase Force
PE	71.8 ± 8.7	303.2 ± 43.5	95.1 ± 4.7*	304.1 ± 3.8	54.5 ± 9.8	289.5 ± 31.4	49.3 ± 9.2	331.5 ± 44.2
PE + l‐NAME	122.3 ± 7.0#	314.9 ± 43.5	107.9 ± 8.0#	363.2 ± 2.6	93.1 ± 6.8	291.8 ± 16.9	96.1 ± 4.5	354.1 ± 24.3
KCl	98.8 ± 1.0	132.8 ± 7.3	97.2 ± 4.2	288.4 ± 35.2*	94.6 ± 31.9	126.2 ± 10.7	99.2 ± 2.5	289.4 ± 22.5*
Treatment	% Relaxation	% Relaxation	% Relaxation	% Relaxation	% Relaxation	% Relaxation	% Relaxation	% Relaxation
ACh	70.2 ± 3.5	98.4 ± 1.5	56.3 ± 8.1 4*ϕ*	98.6 ± 0.6	82.1 ± 2.2	95.5 ± 1.7	93.6 ± 3.67	100.1 ± 1.3
ACh + l‐NAME	19.2 ± 0.7*	87.1 ± 6.9	–14.2 ± 2.0	96.3 ± 10.3	–13.4 ± 4.4	79.9 ± 11.6	–14.7 ± 4.7	90.5 ± 2.9
SNP	93.0 ± 2.6	94.9 ± 3.4	96.3 ± 5.3	99.1 ± 0.84	89.3 ± 3.6	92.4 ± 1.1	90.4 ± 1.4	96.4 ± 1.7
(B)								
Treatment	pD2, (−log EC50)	pD2, (−log EC50)	pD2, (−log EC50)	pD2, (−log EC50)	pD2, (−log EC50)	pD2, (−log EC50)	pD2, (−log EC50)	pD2, (−log EC50)
PE	10.39 ± 0.17	5.7 ± 0.1	9.93 ± 0.17	4.1 ± 0.2	9.98 ± 0.10	5.6 ± 0.1	9.77 ± 0.16	4.3 ± 0.1
PE + l‐NAME	10.54 ± 0.07	5.9 ± 0.2	10.25 ± 0.13	2.7 ± 0.1	10.41 ± 0.08	6.1 ± 0.1	10.27 ± 0.09	2.9 ± 0.1
KCl	1.74 ± 0.09#	1.7 ± 0.0	1.82 ± 0.12#	1.3 ± 0.7*ϕ*	2.15 ± 0.12	1.7 ± 0.8	2.27 ± 0.20	1.4 ± 0.4*ϕ*
ACh	7.25 ± 0.06	8.2 ± 0.3	7.75 ± 0.24	8.7 ± 0.5	7.09 ± 0.21	7.8 ± 0.2	7.27 ± 0.34	8.9 ± 0.3
ACh + l‐NAME	–	7.6 ± 0.2	–	6.9 ± 0.2*ψ*	–	7.7 ± 0.2	–	7.1 ± 0.1*ψ*
SNP	7.48 ± 0.06	7.4 ± 0.6	7.75 ± 0.12	7.7 ± 0.6	7.52 ± 0.16	7.8 ± 0.6	7.27 ± 0.09	7.9 ± 1.0

l‐NAME, l‐Nitro‐Arginine Methyl Ester; SHR, spontaneously hypertensive rats; WKY, Wistar Kyoto rats.

*P < 0.05 versus all other groups, ^#^Significant versus female rats, *^ϕ^P* < 0.05 versus WKY rats, ^*ψ*^*P* < 0.05 versus non‐treated rings.

**Figure 2.**
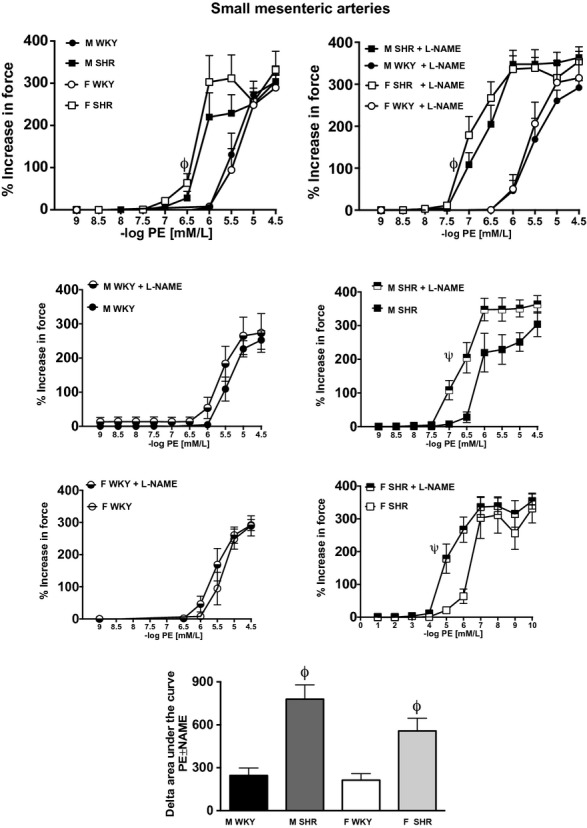
Vasoconstriction induced by PE in small mesenteric arteries from male and female Wistar‐Kyoto rats (WKY) and spontaneously hypertensive rats (SHR) in the absence (A) or presence (B) of l‐Nitro‐Arginine Methyl Ester (l‐NAME). Panel C illustrates the impact of l‐NAME on PE‐induced constriction within each group. Panel D illustrates the ability of NO to attenuate PE‐induced constriction, or the vasoconstrictor buffering capacity of nitric oxide synthase (NOS), which was calculated as the change in the area under the curve in the presence or absence of l‐NAME (% increase in force × mol/L). *ϕ* indicates significant differences in maximal % increase in force versus Wistar‐Kyoto rats (WKY), *P* < 0.05. ^*ψ*^indicates significant differences in maximal increase in force versus nontreated control rings, *P* < 0.05; *N* = 5–6.

In contrast, maximal constriction to PE was comparable between all groups in small mesenteric arteries (Fig. 2A). However, mesenteric arteries from SHR, regardless of sex, had greater sensitivity to PE‐induced constriction than mesenteric arteries from WKY (Fig. 2A and B; effect of sex = NS; effect of strain = *P* < 0.05; interaction = NS). The individual comparisons are depicted in panel 2C. Vasoconstrictor buffering capacity of NOS in small mesenteric arteries was significantly greater in SHR than in WKY (effect of sex = NS; effect of strain = *P* < 0.05; interaction = NS, Fig. 2D).

**Figure 3.**
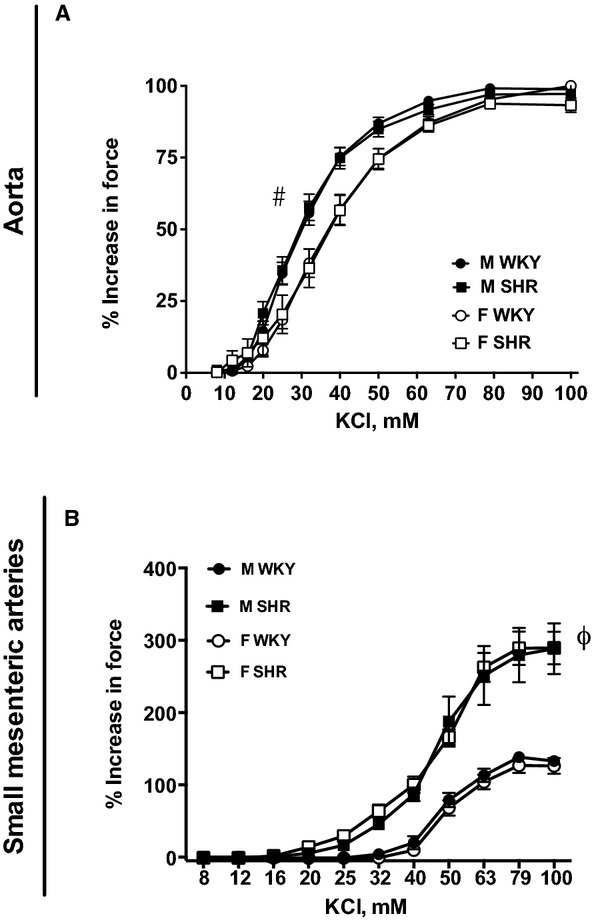
KCl‐induced constriction in aortic (A) and small mesenteric artery (B) rings from male and female Wistar‐Kyoto rats (WKY) and spontaneously hypertensive rats (SHR). ^#^indicates significant differences in maximal % increase in force versus female rats, *P* < 0.05. ^*ϕ*^indicates significant differences in maximal % increase in force versus Wistar‐Kyoto rats (WKY), *P* < 0.05. *N* = 8–12.

Receptor‐independent vascular contraction was also assessed in response to increasing concentrations of KCl. Maximal contraction was comparable in aortic rings from all four groups (Table 1 and Fig. 3A), however, male rats showed greater sensitivity to KCl‐induced vasoconstriction than females regardless of strain. In addition, we found strain differences in KCl‐induced contraction in small mesenteric arteries (Table 1 and Fig. 3B); small mesenteric arteries from SHR displayed greater maximal KCl‐induced contraction and sensitivity compared to WKY regardless of sex.

### Relaxation

**Figure 4.**
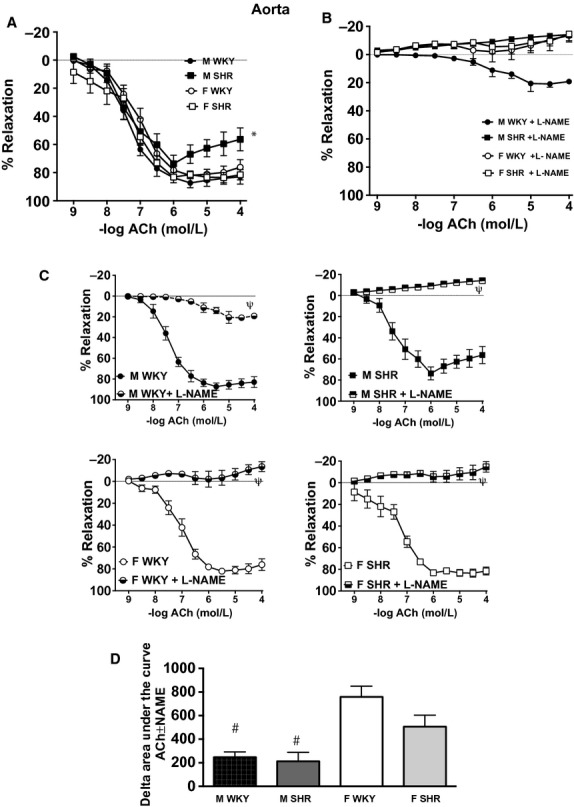
Vasorelaxation induced by ACh in aorta from male and female Wistar‐Kyoto rats (WKY) and spontaneously hypertensive rats (SHR) in the absence (A) or presence (B) of l‐Nitro‐Arginine Methyl Ester (l‐NAME). Panel C illustrates the impact of l‐NAME on ACh‐induced relaxation within each group. Panel D illustrates the ability of NO to attenuate ACh‐induced relaxation, or the contribution of NO to relaxation, which was calculated as the change in the area under the curve in the presence or absence of l‐NAME (% relaxation × mol/L). *indicates significant differences in maximal % increase in force versus all other groups, *P* < 0.05. *^ϕ^* indicates significant differences in maximal % increase in force versus WKY,* P* < 0.05. ^*ψ*^indicates significant differences in maximal increase in force versus nontreated rings, *P* < 0.05. *N* = 5–7.

Endothelial‐dependent vasorelaxation was assessed by performing CCR curves to ACh in aortic rings and small mesenteric arteries from male and female SHR and WKY. Maximal relaxation to ACh was impaired in aorta from male SHR compared with other groups (effect of sex = *P* < 0.05; effect of strain = NS; interaction = *P* < 0.05, Table 1, Fig. 4A). Sensitivity to ACh was comparable between aortic rings isolated from male and female SHR and WKY (Table 1B). l‐NAME preincubation dramatically blunted ACh‐induced relaxation in male WKY and female rats regardless of strain, however, male WKY maintained a dilatory response to ACh in the presence of l‐NAME (Fig. 4B). The individual comparisons are depicted in panel 4C. Vasodilatory buffering capacity of NOS in small mesenteric arteries was significantly greater in female rats compared to male rats (effect of sex = *P* < 0.05; effect of strain = *P* < 0.05; interaction = NS, Fig. 4D).

**Figure 5.**
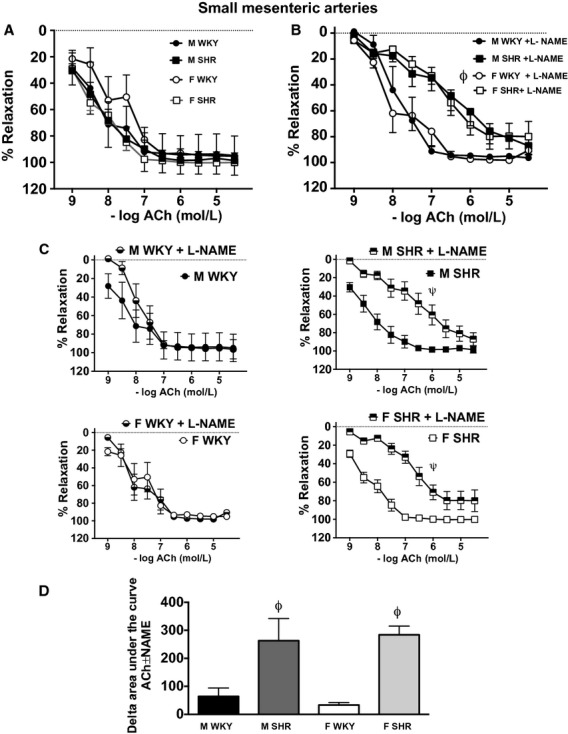
Vasorelaxation induced by ACh in small mesenteric arteries from male and female Wistar‐Kyoto rats (WKY) and spontaneously hypertensive rats (SHR) in absence (A) or presence (B) of l‐Nitro‐Arginine Methyl Ester (l‐NAME). Panel C illustrates the impact of l‐NAME on ACh‐induced relaxation within each group. Panel D illustrates the ability of NO to attenuate ACh‐induced relaxation, or the contribution of NO to relaxation, which was calculated as the change in the area under the curve in the presence or absence of l‐NAME (% relaxation × mol/L). *indicates significant differences in maximal % increase in force versus all other groups, *P* < 0.05. ^*ϕ*^indicates significant differences in maximal % increase in force versus WKY,* P* < 0.05. ^*ψ*^indicates significant differences in maximal increase in force versus nontreated rings, *P* < 0.05. *N* = 5–7.

Maximal relaxation to ACh in small mesenteric arteries was similar in all groups (Table 1 and Fig. 5A). l‐NAME preincubation attenuated ACh‐induced relaxation and sensitivity in mesenteric arteries from SHR compared to WKY (effect of sex = NS; effect of strain *P* < 0.05; interaction = NS, Fig. 5B). The individual comparisons are depicted in panel 5C. Consequently, vasodilatory buffering capacity of NOS in small mesenteric arteries was significantly greater in SHR compared to WKY (effect of sex = NS; effect of strain = *P* < 0.05; interaction = NS, Fig. 5D).

To determine if sex and strain differences in response to ACh were related to vascular smooth muscle sensitivity to NO, endothelial‐independent vasorelaxation was also assessed in response to SNP. SNP‐induced relaxation was similar in aortic and small mesenteric rings from male and female SHR and WKY (Table 1).

### Total NOS activity and expression in vascular tissue

**Figure 6.**
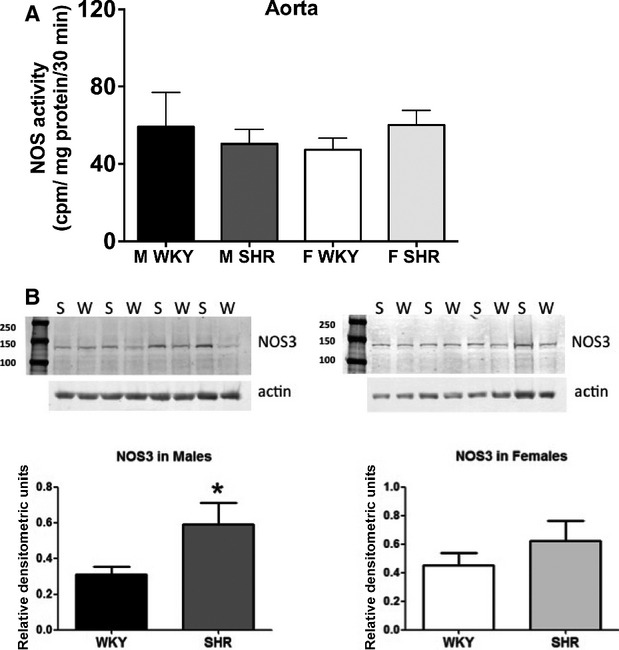
Nitric oxide synthase (NOS) activity (A) and NOS3 protein expression (B) in isolated aorta from male and female Wistar‐Kyoto rats (WKY) and spontaneously hypertensive rats (SHR). The first lane in each representative Western blot indicates the molecular weight marker. *indicates increased protein expression versus male WKY, *P* < 0.05. *N* = 8–9.

To begin to gain mechanistic insight into observed changes in vascular function, NOS enzymatic activity and NOS3 protein expression were assessed in aorta and mesenteric arteries of male and female SHR and WKY. Total NOS enzymatic activity was comparable in aorta from male and female SHR and WKY (Fig. 6A). Although NOS activity was comparable, male SHR had greater NOS3 protein expression than WKY; aortic NOS3 protein expression was similar in female SHR and WKY (Fig. 6B).

**Figure 7.**
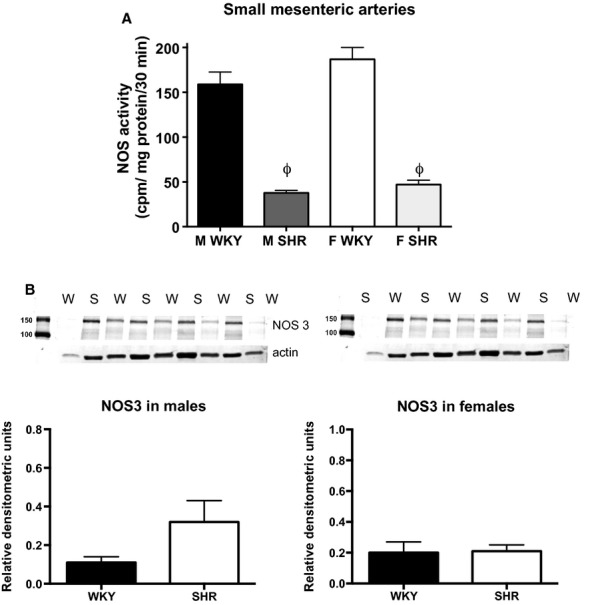
Nitric oxide synthase (NOS) activity (A) and NOS3 protein expression (B) in the mesenteric arterial bed from male and female Wistar‐Kyoto rats (WKY) and spontaneously hypertensive rats (SHR). The first lane in each representative Western blot indicates the molecular weight marker. ^*ϕ*^indicates significant difference from M WKY,* P* < 0.05. *N* = 8–9.

In contrast, NOS activity was significantly greater in small mesenteric arteries from WKY compared to SHR (Fig. 7A); NOS activity was comparable between the sexes in both strains. Consistent to what was observed in the aorta, male SHR tended to have greater NOS3 expression in small mesenteric arteries than male WKY and there were no significant difference in NOS3 expression in female SHR and WKY (Fig. 7B).

## Discussion

Loss of vascular NO results in impaired vasodilation and enhanced vasoconstriction which have been suggested as a common pathway in the pathogenesis of cardiovascular disease (Giles et al. 2012). The current study examined the impact of both sex and blood pressure status on vascular function. At the age studied in the current manuscript, SHR have established hypertension and blood pressure is higher in the males than in the females (Sullivan et al. 2007; Tipton et al. 2014). In addition, we studied both a large conduit vessel, the aorta, and a small vessel to determine if the effects of sex and hypertension are dependent on artery type. The central findings of the current study are that male SHR exclusively exhibit altered aortic function and decreases in the NOS contribution to vascular tone, while in mesenteric arteries SHR of both sexes had enhanced vascular responses and increases in the NOS‐sensitive component. Based on the central role played by small arteries in the regulation of blood pressure, we hypothesize that SHR exhibit a compensatory increase in NOS in response to increases in blood pressure to help maintain small artery function. Additional studies are required to test this hypothesis since neither sex nor strain altered NOS enzymatic activity, and NOS3 protein expression was not increased in SHR compared to WKY. Regardless, there are sex and strain differences in aortic versus mesenteric arterial function and in the ability of NOS to modulate vascular function.

PE‐induced contraction and basal NOS‐mediated attenuation of contraction were comparable in female SHR and WKY, although impaired in male SHR relative to all other groups. Basal NO availability in male and female WKY and stroke‐prone SHR (SHRSP) has also been examined using l‐NAME in PE‐constricted aortic rings (McIntyre et al. 1997). In contrast to our findings, basal NO availability was reduced in aortas from SHRSP of both sexes compared to WKY, although females of both strains exhibited greater NO levels than males. Additional studies reported that aortic rings from male and female SHRSP exhibited greater PE‐induced constriction than rings from WKY and confirmed decreases in basal NO in both carotid arteries and aorta in SHRSP versus WKY (Dowell et al. 1999; Kerr et al. 1999). The discrepancy in these studies relative to ours may be related to SHR/SHRSP strain differences. Indeed, consistent with our study, aortic strips from male SHR exhibited greater increases in active stress than strips from female SHR and this was accompanied by greater increases in calcium influx (Crews and Khalil 1999a,b). Aortic segments from male SHRSP also display greater contraction to calcium and increased activation of STIM1 and Orai1 relative to females, therefore, sex differences in calcium handling and signaling in aorta from female SHR may contribute to the maintenance of vascular function (Giachini et al. 2012) relative to males. Alternatively, 12‐week‐old male SHR have a higher blood pressure compared to females. Therefore, while the mechanism responsible for the alterations in aortic function in male SHR is unknown, higher blood pressure may also contribute.

Unlike aorta, small arteries play a physiologically key role in the regulation of blood flow, and ultimately contribute to blood pressure. Others have reported a greater PE‐induced contraction in small mesenteric arteries from SHR compared to WKY (Nyborg and Bevan 1988; Husken et al. 1994; Toot et al. 2011). In our study, we found an increased sensitivity to PE only in SHR, with similar maximal constriction in all groups. In addition, despite decreased basal NO in aorta from male SHR, SHR of both sexes exhibited an increase in NOS‐mediated attenuation of PE‐induced contraction with no effect in WKY, suggesting greater basal NO in small arteries from hypertensive animals to attenuate PE‐induced constriction. There were no sex differences in either strain in PE‐included constriction or the impact of l‐NAME, consistent with our previous publications in SHR and normotensive F344 Brown‐Norway rats (Sullivan et al. 2004, 2009).

To further determine if sex/strain differences in PE‐induced contraction were due to differences in the contractile machinery, we assessed KCl‐induced contraction as well. KCl‐induced maximal vasoconstriction in aortic rings was similar in male and female WKY and SHR; however, rings from male rats displayed greater KCl sensitivity than from females regardless of strain. Our data suggest that males have intrinsically increased contractile function compared to females. Consistent with our data, it has been reported that in freshly isolated cells from thoracic aorta that contraction triggered by calcium as well the basal cytosolic calcium levels are reduced in female rats regardless of the strain (Crews et al. 1999; Murphy and Khalil 2000). These data further suggest, however, that the sex/strain differences in PE‐induced constriction are not the result of innate differences in the contractile machinery, but rather reflect differences in *α*‐adrenergic‐induced contraction. In contrast, SHR of both sexes exhibited greater maximal contraction to KCl compared to WKY. While maximal contraction to PE was not greater in SHR compared to WKY, there was an increased sensitivity, suggesting both alterations in calcium handling and overall contraction with the development of hypertension as well as sex and strain differences in *α*‐adrenergic‐induced contraction.

Endothelial dysfunction is a key factor in the development of cardiovascular disease in men and women (Ballerio et al. 2007; Skaug et al. 2013) and endothelium‐dependent vascular relaxation to ACh is impaired in patients with hypertension and in various experimental models of hypertension (Ballerio et al. 2007; Gamboa et al. 2007). Consistent with male SHR exhibiting impaired aortic basal NO release, male SHR also exhibited an attenuation of ACh‐induced relaxation in the aorta. While only male SHR exhibited a decrease in maximal relaxation to ACh, aortic rings from males of both strains exhibited less NOS‐mediated relaxation compared to females. In SHR, this was due to a decrease in overall relaxation, while male WKY exhibited a degree of NOS‐independent relaxation not seen in the other groups. This was an interesting finding since ACh‐induced relaxation is almost exclusively dependent on NO (Furchgott and Zawadzki 1980; Kauser and Rubanyi 1994). Future studies will examine the mechanism responsible for the residual relaxation to ACh in the presence of l‐NAME in male WKY, yet regardless, males of both strains exhibited less agonist‐stimulated NO release. While this is consistent with numerous reports that females have greater NO levels than males (Kauser and Rubanyi 1994; Huang et al. 1998), it has previously been reported that aortic rings from SHR of both sexes have impaired ACh relaxation compared to WKY (Silva‐Antonialli et al. 2000). However, in that study, SHR were older and males and females were combined in a single group and not studied separately. Therefore, the decrease in function could have been “driven” by aortic segments from the males. In a separate study, aortic rings from male and female SHRSP exhibited greater sensitivity to ACh‐induced relaxation compared to WKY, although there were no apparent sex differences (Dowell et al. 1999). It is tempting to speculate that impaired ACh‐induced relaxation exclusively in male SHR contributes to greater increases in blood pressure or end‐organ damage compared to females. However, since the aorta is not an artery that regulates blood pressure, additional studies also included small mesenteric arteries.

Interestingly, ACh‐induced relaxation was comparable in all groups, although SHR exhibited greater NOS‐mediated attenuation of the dilatory response compared to WKY regardless of sex. l‐NAME had minimal impact on ACh‐induced relaxation in WKY. In small mesenteric arteries, ACh‐induced dilation is mediated by NO, dilatory prostaglandins, and EDHF (Matrougui et al. 1997; Takamura et al. 1999; Makino et al. 2000). Therefore, our data suggest a shift in the balance of these dilatory pathways to maintain relaxation with hypertension, such that the NOS‐mediating component is increased, potentially in response to increases in blood pressure to help limit further increases in pressure and vascular dysfunction. In contrast to our findings, relaxation to ACh in norepinephrine‐precontracted mesenteric rings was attenuated in both female and male SHR compared to WKY (Kauser and Rubanyi 1995), however, the lack of a sex difference in ACh‐induced relaxation is consistent with previous publications from our group (Sullivan et al. 2004, 2009). Others have shown that the relative role of NO in the endothelium‐dependent and independent relaxation was higher in female than in male SHR. However, the size of the mesenteric arteries studied was not indicated in this experiment therefore the results may be more relevant to our findings in the aorta (Kahonen et al. 1998).

To gain insight into the molecular mechanisms by which sex and strain impact vascular function, NOS activity and NOS3 expression were assessed. Despite both sex and strain differences in the relative contribution of NOS to impact vascular function, NOS enzymatic activity was comparable among all groups. Moreover, NOS3 protein expression was greater, not less, in aorta from male SHR compared to male WKY and SHR did not have greater NOS3 expression in the mesenteric arterial bed. Therefore, neither our measurements of NOS activity nor NOS3 expression explained the sex and strain differences in vascular function. It should be noted that NOS activity was measured via an enzymatic assay in the presence of excess amounts of all of the necessary cofactors and does not necessarily reflect in vivo NOS activity or NO production. In a separate study, direct in vitro measurements of NO in mesenteric arteries showed unchanged NO release, but significantly greater NO decomposition by reactive oxygen species (ROS) (Tschudi et al. 1996). Indeed, the attenuated basal NOS component in aorta from male SHR likely reflects differences in ROS production. NO bioavailability is determined not only by NOS activity and expression but also by scavenging of NO by superoxide (Kerr et al. 1999) and male SHR have greater levels of oxidative stress than female SHR (Yanes et al. 2005; Iliescu et al. 2006, 2007) and WKY (Kerr et al. 1999). Therefore, it is likely that greater levels of oxidative stress in SHR compared to WKY, particularly in males, contributes to the decrease in NOS buffering capacity in aortic tissue. The potential even exists that maintained NOS activity in SHR reflects uncoupled NOS activity, in aortic rings from male and female SHRSP treatment with l‐NAME decreases in superoxide levels (Kerr et al. 1999), directly implicating NOS as a source of ROS in the vasculature. It should also be noted that total NOS activity was measured, not isoform‐specific activity. In addition to NOS3, NOS1 in particular is also found in the vasculature and we have previously shown that NOS1 typically acts as a break to increases in contraction (Sullivan et al. 2002) in isolated mesenteric arteries. Therefore, there could be isoform‐specific changes in NOS activity, or NOS1 protein, which were not assessed in the current study.

It is tempting to speculate that in our study, the reduced NOS buffering capacity to PE constriction may contribute to differences in pulse pressure in a sex and strain‐dependent manner. Although not measured in the current study, it has previously been shown that male SHR show significantly higher pulse pressure than female SHR while pulse pressure in the normotensive WKY rats is comparable between males and females (Chamiot‐Clerc et al. 2001; Maris et al. 2005). Furthermore, SHR had greater pulse pressure than WKY rats when compared for each gender. This may reflect sex and strain differences in sympathetic nervous system activity, cardiac contractility, stroke volume, vasomotor tone, and/or vascular compliance.

In conclusion, we propose that SHR exhibit a functional increase in ACh‐induced NO production in small arteries to compensate for hypertension‐induced vascular damage. This study provides further evidence regarding sex differences in the mechanism regulating vascular tone in WKY and SHR.

## Conflict of Interest

None declared.
